# Significantly increased low shear rate viscosity, blood elastic modulus, and RBC aggregation in adults following cardiac surgery

**DOI:** 10.1038/s41598-018-25317-8

**Published:** 2018-05-08

**Authors:** Yi-Fan Wu, Po-Shun Hsu, Chien-Sung Tsai, Pin-Cheng Pan, Yeng-Long Chen

**Affiliations:** 1 Institute of Physics, Academia Sinica, Taipei, Taiwan; 2 Department of Chemical Engineering, National Tsing-Hua University, Hsinchu, Taiwan; 3 Division of Cardiovascular Surgery, Department of Surgery, Tri-Service General Hospital, National Defense Medical Center, Taipei, Taiwan

## Abstract

Open heart surgeries are common for treating ischemic and heart valve disease. During cardiac surgery, cardiopulmonary bypass (CPB) can temporarily take over the function of heart and lungs. However, elevated red blood cell (RBC) aggregation may lead to the common side-effects such as microinfarction. We investigated blood physical properties changes and the correlation between blood microstructure, viscoelastic response and biochemical changes following surgery with CPB. We examined shear-rate dependent blood viscosity, elasticity and RBC aggregate size in the pre-surgery disease state, post-surgery state and long-term recovery state of cardiac surgical patients. Within a week following surgery, the patient hematocrit was significantly lower due to CPB. Despite lower RBC concentration, the RBC aggregate shape became larger and more rounded, which is correlated to the elevated plasma fibrinogen related to systemic inflammatory response. During the same period, the hematocrit-adjusted low shear rate viscosity increased significantly, as did the yield stress, indicating more solid-like behavior for blood. Six months to one year later, all the physical and biochemical properties measured returned to baseline.

## Introduction

Cardiovascular disease (CVD) is the most common cause of death worldwide, with the total number of CVD deaths increasing by 40% between 1990 and 2013^1^. Cardiac surgical treatments for ischemic heart disease and heart valve disease have been effective in extending the life expectancy of patients. However, the surgical treatment causes side-effects that include systemic inflammation^2^, thrombotic responses, endothelial cell activation^3^ and tissue damage^4^. Other complications such as cerebral ischemia and microinfarction could result in symptoms of impaired cognition or memory deficits, and they have been the focus for several recent studies^5–8^. These symptoms may be related to reduced oxygen delivery due to the increased blood viscosity and red blood cell (RBC) aggregation post-surgery. Previous studies have suggested that RBC aggregation can lead to many adverse effects at low shear rates ($\dot{\gamma }$ = 0.2 to 1.4 s^−1^, $\dot{\gamma }$ = mean flow velocity/vessel radius) in the cerebral arterioles, post-capillary venules, and near flow stagnation points^9^.

Chien and coworkers first accurately quantified altered blood viscosity with pathophysiological research^10,11^. Blood viscosity is now a known indicator of CVD^12^, peripheral arterial disease^13,14^, arterial hypertension^15^, diabetes mellitus^16^, high cholesterol and triglyceride levels^17^, and sickle-cell anemia^18^. In addition, there is a strong correlation between elevated RBC aggregation and inflammation^19,20^. Although whole blood exhibits both viscous and elastic properties, the quality of viscoelasticity as an indicator of CVD has received less attention in the literature.

Many previous hemo-rheological studies focused on blood viscosity at high shear rate (>150 s^−1^)^13,14,21–24^ comparable to arterial flow as disease indicators. Several studies have characterized healthy human blood viscosity at various shear rates^25–29^. For CVD patients, Jan *et al*. found that for patients who suffered acute myocardial infarction, changes in the low shear rate viscosity were more significant^10^. Factors such as temperature, plasma protein concentration, hematocrit, RBC shape, RBC aggregation, deformability, and shear force affect blood viscoelasticity^30^. Difficulties in rheological characterization lie in the dynamic dependence of RBC aggregation and deformation on the applied shear rate. At low shear rates (<10 s^−1^), RBC aggregation results in higher viscosity and elasticity^31^. At high shear rates, blood viscosity varies with RBC deformation. A reliable method for evaluating blood rheology may improve disease diagnosis, evaluation of treatment effectiveness, and recovery monitoring of CVD patients. This study investigated a wide range of pathological rheological properties from high to low shear rates, relevant to flow in the micro-capillaries, artery, ascending aorta, vena cava, and cerebral arterioles.

Cardiac surgeries can induce abnormal blood rheological characteristics, which may be related to lung dysfunction and endothelial damage^32^. For decades, many studies have focused on complications such as cerebral ischemia, memory deficits cognitive loss and neurologic injury in post-surgical patients^5,6^. To reduce these post-operative complications, hemodilution is applied during surgeries using cardiopulmonary bypass (CPB) to lower blood hematocrit and viscosity, thus increasing blood fluidity during and after the cardiac surgical procedure. Since many studies have shown that the diuretics, anti-platelet and anti-coagulation drugs do not influence the HCT or viscosity^33,34^, hemodilution has become more apparent to be the direct cause of the side-effects. Although hemodilution reduces elevated viscosity, the side-effects are not entirely known. In several studies over the past decade, excessive hemodilution has been linked with an increase in post-operative mortality, renal deficiency^35^ and stroke^36^. This study analyzed blood rheological characteristics and RBC aggregation in cardiac surgical patients before and after surgery. We also correlated the physical characteristics with the relevant biochemical protein concentrations. The results may assist in the evaluation of post-surgical treatments, recovery, and differentiate between heart disease patients and normal subjects.

## Materials and Methods

Patient recruitment was coordinated by the Division of Cardiovascular Surgery of Tri-Service General Hospital (TSGH). After receiving approval of the study protocol from the Institutional Review Board of TSGH (TSGHIRB- 2-103-05-092), written informed consents were obtained from participants or their legal designates. All procedures of the studies involving human participants were performed in accordance with the guidelines and regulations of the institution.

**Figure 1:**
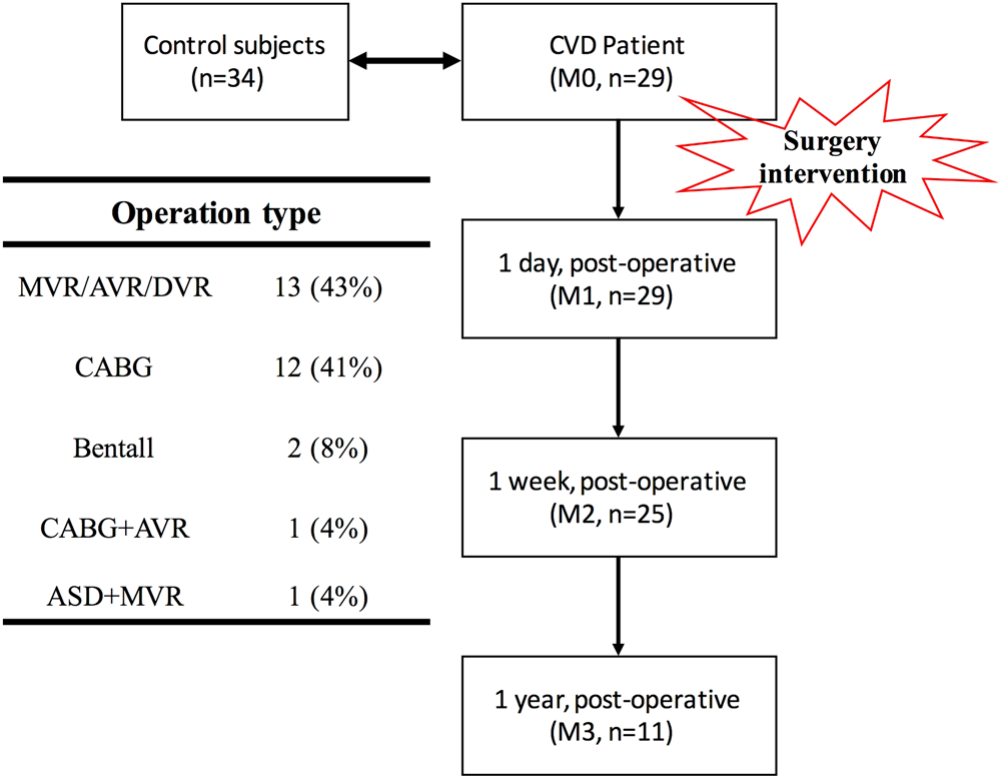
Sample collection flowchart for surgical patients and elective CVD surgery type. CVD surgery type - CABG: Coronary Artery Bypass Grafting; MVR/AVR/DVR, Mitral/Aortic/Double Valve Replacement; ASD, Atrial Septal Defect.

All the subjects were native Taiwanese, and none of the subjects exercised or consumed food for at least an hour before each experiment. Surgical patients were required to fast overnight (at least 8 hours) before the surgical procedure. All blood samples were collected between 8 and 10:30 AM in the same morning. Samples were collected from 29 cardiac patients (22 males/7 females; age range between 25 and 75 years (with approximate mean age of 63 years) undergoing elective cardiac surgical procedures, which are listed in Fig. 1. All surgical patients required CPB. The control group (Control) consisted of 34 normal people without CVD-related diseases or diabetes.

### Intervention

Open-heart surgery was performed at the TSGH after overnight fasting. Sarns pumps (Sarns, Ann Arbor, MI) and bubble oxygenators (Terumo, Tokyo, Japan) were used during the cardiac surgical procedure for extracorporeal circulation with CPB. The oxygenators were primed with 1000 ml Voluven (starch fluid) and 250–500 ml packed RBCs from a blood bank (not from the patient himself/herself). The mean total bypass time (pump time) for these patients was 124 minutes, with a mean total cross-clamping time of 83 minutes.

### Clinical and Rheological Measurements

In these 29 patients, blood samples were taken before the surgical procedure (M0) as well as on the first day (M1), one week (M2), and between 6 months to one year (M3) after surgery. Physiological parameters measured included the heart rate (HR), systolic blood pressure (SBP), and diastolic blood pressure (DBP). Blood samples were collected into Vacutainers (BD, Franklin Lakes, NJ, USA) containing ethylenediaminetetra-acetic acid (EDTA, an anticoagulant). Vacutainers with sodium-citrate were used for the fibrinogen (Fib) measurement. All rheological studies were performed within 5 hours after the blood draws^37^. Additionally, inflammation and tissue damage indicators, such as C-reactive protein (CRP) and Fib concentrations were measured.

Rheology measurements were made with Physica rheometer MCR 501(Anton-Paar, Graz, Austria) with concentric-cylinder geometry (CC-MS with 425 μm gap size) under automated dynamic shear. The fluid stress response to an induced strain was measured. Whole blood viscosity (WBV), plasma viscosity (PV), and viscoelastic moduli were calculated using the stress-strain relation^26^. CC-MS was chosen for its high sensitivity in low viscosity fluid measurements due to its high surface area and minimum sample evaporation. Further, it also a standard rheology geometry (ISO-3219). Our hemo-rheological measurements followed the protocol outlined by Baskurt^38^. Whole blood was gently agitated to homogenize the sample before loading. The bob connected to the motor rotated inside the cup, inducing shear flow within the agitated blood, which was kept at 37 °C. Experiments were performed at the native hematocrit of the blood sample.

We first performed unidirectional shear rate sweep measurements by increasing the shear rate from 0.1 s^−1^ to 1000 s^−1^ in steps, and then with the shear rates decreasing from 1000 to 0.1 s^−1 ^^39^. For blood plasma, PV was only measured at 532 s^−1^ due to its relative independence to shear rate. We also performed oscillatory shear experiments to measure the elastic (G’) and viscous (G”) moduli under strains (deformation/gap size, γ) from 1 to 1000% in oscillatory frequency (ω) of 1 rad/s, corresponding to shear rates between 0.01 to 10 s^−1 ^^28,29,40^. To reduce the effect of RBC sedimentation, each measurement time was less than 20 minutes^41^. Increasing G’ reflects non-Newtonian energy storage elasticity, whereas increasing G” reflects flow dissipation.

### RBC aggregation measurement

In addition, blood microstructure was characterized at rest by optical microscopy (Nikon E100; Nikon Corp, Japan). Each blood sample was mildly centrifuged for 10 min at 2000 rpm, following which RBCs were re-suspended in autologous plasma at 1% hematocrit^38^. The suspension was placed into a narrow-gapped (up to 100 μm) microscopic chamber and images were taken within 5 minutes following preparation, by charge-coupled device (CCD) camera using a 10× objective. Five distinct regions, each over an area of 427 × 285 μm, were imaged for each sample. Images were analyzed using ImageJ to determine the average aggregate size (AAS)^25,42^ and the aggregate shape parameter (ASP)^43,44^. The ASP is defined as the ratio between the projected area (A) and the perimeter (P) of the aggregate, ASP = 4πA/P^2^. We calculated the aggregate size by comparing the area of columnar rouleaux aggregates with the projected RBC lateral area (≈12.25 µm^2^ and ≈37 pixels at 10X magnification). The total average is performed with 5 images per sample. In each image, there were at least 15 aggregates. We assume that the RBC lay such that the disc-plane is perpendicular to the imaging plane in order to estimate the aggregate size. This requires us to discount RBC and aggregates that lay top-down. By trial-and-error, we found that the criteria for choosing columnar aggregates is ASP <0.2 and >0.9. Approximately 11% of the aggregates in the patient samples and 18% in the Control samples are not counted with this criterion.

### Data Analysis

Blood viscosities were measured from 0.1 to 1000 s^−1^. We picked four specific shear rates (298/s, 26.4/s, 4.28/s and 0.38/s) that are representative of various circulatory flow conditions to compare the relative viscosity change^45^. These shear rates correspond to flow conditions in the cerebral arterioles (0.2~1.4 s^−1^), vena cava (~60 s^−1^), arteries (~190 s^−1^), and micro-capillaries (~1000 s^−1^), respectively. The native-HCT WBV (*η*_*a*_) and PV (*η*_0_) at each shear rate were averaged over all subjects in each group. WBV was measured at the native HCT and scaled by the relative viscosity *η*_rel_ = *η*/*η*_0_ to account for individual differences in PV. Differences in individual subject hematocrit can be accounted by the hematocrit dependence^46^ given by *η*_rel(45)_ = [*η*_rel(HCT)_]^45/HCT^, which finds the HCT-adjusted blood viscosity for comparison at 45% HCT^47^.

We also compared the relative individual WBV changes of the post-surgical groups (M1, M2, M3) with the pre-surgical measurement (M0) to elucidate post-surgical effects and account for individual physiological differences. All quantities are presented as the mean ± standard deviation (SD) or median with inter-quartile range (if not normally distributed). The differences were characterized using the P-value from the Student’s paired t-test, Mann–Whitney U-test or Wilcoxon’s test, where appropriate. The association between any two measurements was examined using Spearman’s rank correlation *r*. The statistical analysis was performed using IBM SPSS, version 22.0 (IBM Co., Armonk, NY, USA).

## Results and Discussion

### Biochemical changes

Blood tests were performed on the same samples in order to obtain the individual physiological parameters (SBP, DBP, HR, and BMI), as shown in Table 1. The patient group sample size declined from M0 to M3 due to patient discharge a week after the surgery and fewer returning patients for the 6 months to a year follow-up after surgery.

**Table 1:** Subjects’ Physiological Parameters.

Variable	Group				
	Control	M0	M1	M2	M3
n	34	29	29	25	11
Males/n	22/34	22/29	22/29	20/25	10/11
Age, years	26.2 ± 9.7	63.2 ± 13.1^‡^	63.2 ± 13.1	64.2 ± 14.0	65.7 ± 10.1
BMI, kg/m^2^	22.2 ± 1.9	24.2 ± 3.4^‡^	24.2 ± 3.4	24.0 ± 3.5	
Systolic Blood Pressure, mm-Hg	108.5 ± 12.3	123.8 ± 18.3^‡^	144.0 ± 21.9	115.0 ± 13.3	144.3 ± 20.0
Diastolic Blood Pressure, mm-Hg	73.12 ± 9.9	72.8 ± 11.8	73.7 ± 8.7	69.7 ± 9.3	78.4 ± 8.9
Heart Rate, beats/min	68.9 ± 12.0	75.3 ± 9.6	78.2 ± 10.7	82.8 ± 13.2	71.9 ± 10.6
Fibrinogen, mg/dl	257.94 ± 14.23	409.00 ± 23.54^‡^	510.19 ± 36.31^*^	626.90 ± 25.78^†^	373.02 ± 15.02
Hematocrit**, %**	41.90 ± 2.69	39.43 ± 3.02^‡^	30.69 ± 2.58^†^	30.41 ± 2.63^†^	40.34 ± 4.28
C-Reaction Protein, mg/l	0.05 (0.04–0.15)	0.84^‡^ (0.18–1.21)	5.67^†^ (3.06–6.98)	4.00^†^ (2.59–4.98)	
RBC counts, 10^6^/µl	4.97 ± 0.53	4.64 ± 0.74	3.71 ± 0.41^†^	3.57 ± 0.47^†^	4.34 ± 0.32
Hemoglobin, g/dl	14.46 ± 1.47	12.58 ± 1.93^‡^	11.19 ± 5.31	10.14 ± 1.25^†^	13.63 ± 1.63^†^

Physiological parameters (mean ± SD) of subjects. “*” and “^†^”indicate *P* < 0.05 and *P* < 0.01, respectively, when compared with the Pre-surgical value (M0) by the two-tailed paired t-test. “^‡^”indicates *P* < 0.01 compared with the Control by two-tailed t-test. Age (*P* < 0.01), BMI (*P* < 0.01) and systolic blood pressure (*P* < 0.01) were significantly larger at M0 than in the Control (^‡^*P* < 0.01 by two-tailed t-test). For the M3 group, CRP was not available.

BMI for M0 was significantly greater than the Control (*P* < 0.01). SBP increased slightly from 123.8 mmHg in M0 to 144 mmHg in M1 (*P* < 0.1). DBP was not significantly different between M0 and M1. Fib and CRP are protein factors commonly sensitive to RBC aggregation, as they both correlate with infection, trauma, and inflammation^48^. The Fib and HCT levels in the Control were approximately 258 ± 14 mg/dl and 42 ± 3% respectively; these results are similar to the values of 245 ± 30 mg/dl and 41 ± 4% reported previously^49^. Fib was generally higher for the patients than the Control. Fib was elevated immediately post-surgery (M1 and M2) compared to M0. The CRP level showed the greatest percentage change post-surgery, with more than a 400% increase for M1 and M2. In M3, the Fib level  was lower than at M0, M1 and M2.

On the other hand, HCT fell significantly to approximately 30% (*P* < 0.01) for M1 and M2, compared to approximately 45% for Control and M0. This was due to hemodilution during CBP to reduce RBC aggregation and blood viscosity. RBC counts and hemoglobin concentration showed similar trends as the HCT. The HCT at M3 was not significantly different from the Control.

### RBC aggregate microstructure

**Figure 2:**
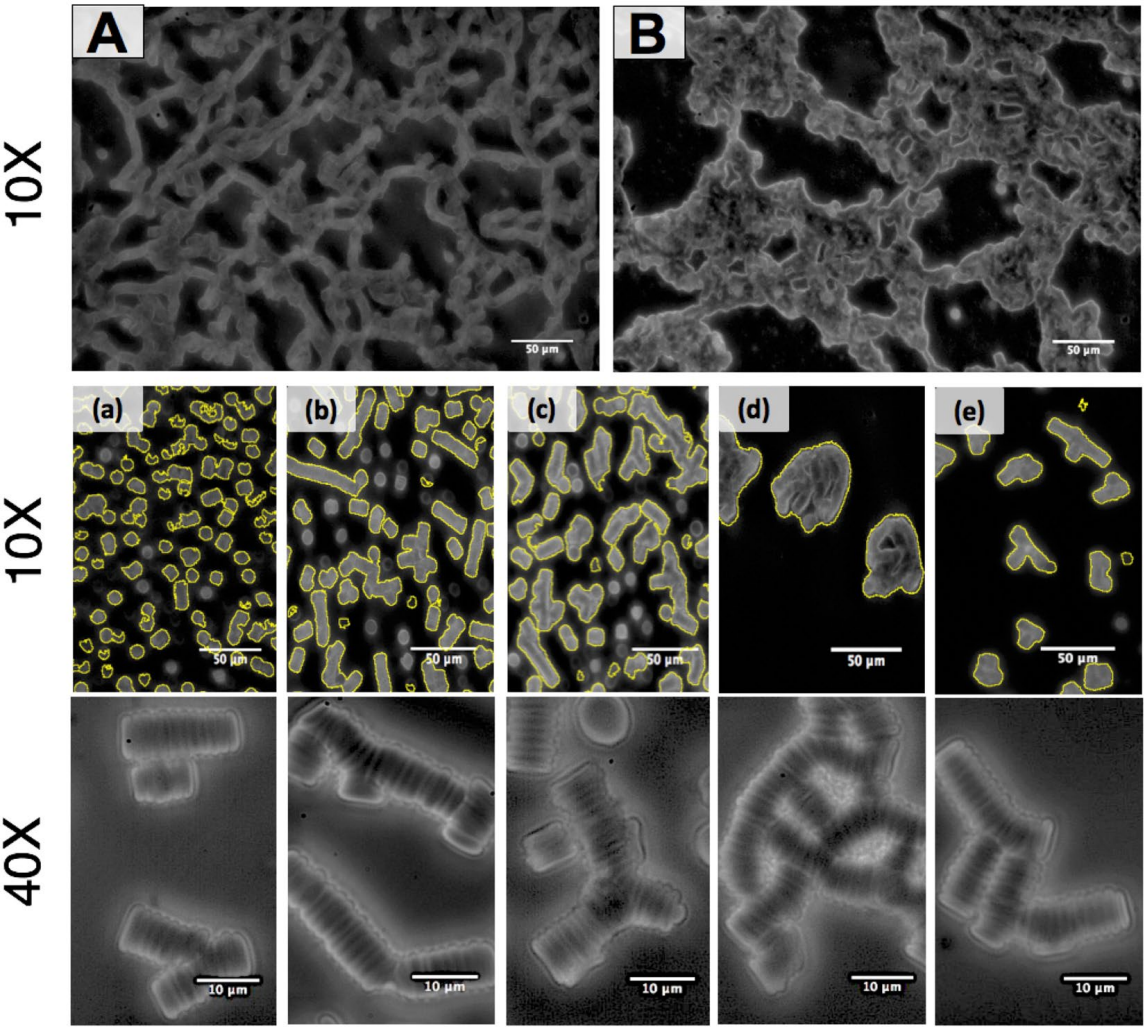
(Top) Two cropped images showing inter-RBC microstructure of RBC aggregates at the native hematocrit for  (**A**) healthy donor (**B**) cardiac patient. (Middle and Bottom) Diluted RBC suspension in autologous plasma at 1%. Left to right: Control, M0, M1, M2, and M3. Yellow outlines indicate selected aggregrate boundaries.

Blood viscoelasticity has clear dependence on RBC aggregate microstructure and its response to shear flow. RBC aggregate microstructure was characterized by optical microscopy at 10X magnification. For the Control group, the RBC aggregate microstructure is a network of rouleaux columns with large voids distributed within the network, as shown in Fig. 2A. For blood from the cardiac patient, the columnar rouleaux structure is not found (Fig. 2B). Rather, the RBCs appear to aggregate in a random, rounded fashion. This could be attributed to stronger inter-RBC attraction due to higher plasma protein concentrations.

To better characterize aggregate size and shape, we diluted the samples to 1% HCT. The columnar rouleaux structures are prominent for Control (Fig. 2a) and M0 (Fig. 2b), while more rounded aggregates are found for M1 (Fig. 2c) and M2 (Fig. 2d). Table 2 shows that the AAS increased steadily for M0, M1, and M2 from 15.86 to 27.25 cells per aggregate. However, no statistically significant difference in AAS was found between M3 and M0. Since only 11 patients out of the 29 patients returned for the long-term follow-up measurement (37.9%), the comparison is not conclusive. The shape of the aggregate also became less anisotropic as the ASP increased from 0.47 (M0) to 0.55 (M2). These trends indicate stronger adhesion between RBCs, likely due to increased aggregation factors such as Fib and CRP. Increased Fib concentration is known to increase inter-RBC attraction and could result in different aggregate structures. Weaker RBC attraction would allow for more thermal motion to form rouleaux with other RBCs, while strong attraction would lead to strong physical bonds that do not break with thermal motion and random aggregates. The observed large randomly connected networks corresponds to the low shear rate elastic response of M0, M1, and M2.

**Table 2:** Hemorheological and Aggregate Properties.

	Control (n = 34)	M0 (n = 29)	M1 (n = 29)	M2 (n = 25)	M3 (n = 11)
Native-HCT Viscosity, mPa • s 0.38 s^−1^	42.08 (37.79–49.13)	62.40^‡^ (38.15–74.13)	50.80 (38.00–60.55)	62.00 (52.80–69.00)	45.42 (38.31–52.79)
4.28 s^−1^	11.07 (9.02–12.39)	12.25 (10.38–14.15)	10.20 (8.00–12.20)	10.46 (8.99–15.60)	9.39 (8.84–13.03)
298 s^−1^	4.06 (3.63–4.43)	4.04 (3.65–4.26)	3.66 (3.07–4.15)	3.64 (3.32–4.37)	3.76 (3.58–4.15)
HCT-adjusted Viscosity 0.38 s^−1^	41.53 (33.35–49.30)	61.75^‡^ (44.51–84.05)	191.92^†^ (105.26–363.28)	217.98^†^ (125.13–394.74)	54.13 (38.80–76.49)
4.28 s^−1^	9.47 (7.31–11.26)	10.99 (9.33–12.96)	14.04^†^ (11.13–21.04)	17.37^†^ (11.74–28.69)	10.78 (8.04–11.28)
298 s^−1^	3.27 (2.82–3.66)	3.05 (2.58–3.75)	3.69 (3.01–4.19)	3.39 (2.94–4.16)	3.03 (2.77–3.59)
Plasma Viscosity, mPa • s 532 s^−1^	1.35 ± 0.17	1.56 ± 0.23^‡^	1.57 ± 0.31	1.74 ± 0.23*	1.39 ± 0.22
Rheological Response γ = 10% (shear rate = 0.1 s^−1^)	G’ < G”, liquid-like	G‘ > G”, gel-like	G’ > G”, gel-like	G‘ > G”, gel-like	G’ < G”, liquid-like
Crossover point ( Critical Strain γ*), %	X	14.88 (6.15–22.3)	18.20* (12.35–31.60)	46.4 (23.35–86.55)	5.66 (4.50–12.73)
Microscopy AAS	12.39 ± 2.51	15.86 ± 3.25^‡^	19.75 ± 5.77^†^	27.25 ± 7.60^†^	17.24 ± 4.72
ASP	0.37 ± 0.11	0.47 ± 0.14^‡^	0.53 ± 0.09*	0.55 ± 0.09*	0.52 ± 0.12

Mean or median values for hemorheological and aggregation Parameters. “*” and “^†^”indicate *P* < 0.05 and < 0.01 respectively, compared with the pre-surgical value (M0) by the two-tailed t-test or the Wilcoxon’s test. “^‡^”indicates *P* < 0.01 compared with the Control by two-tailed t-test or U Mann-Whitney test.

### Blood viscoelasticity pre- and post-surgery

**Figure 3:**
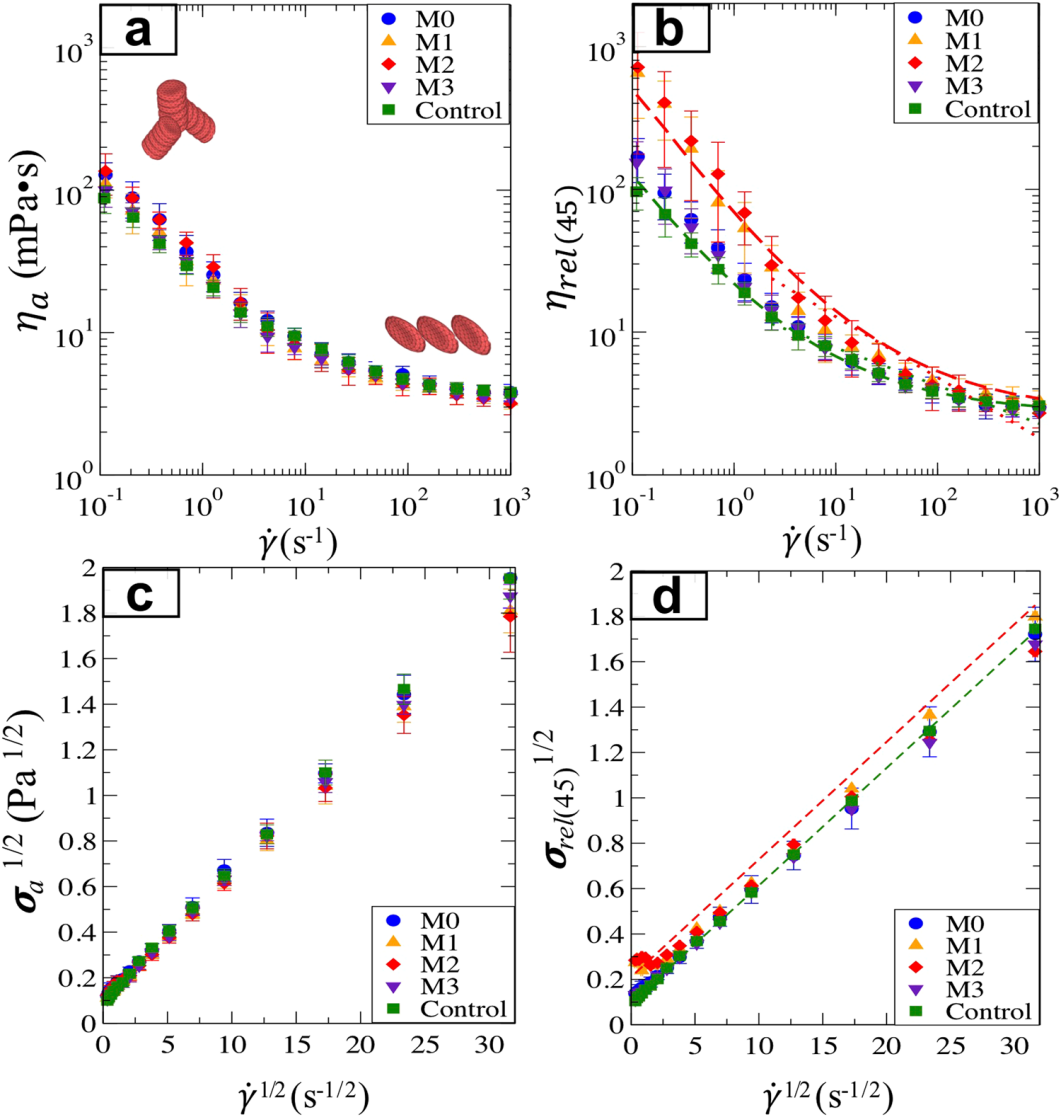
(**a**) Native-HCT blood viscosity. (**b**) HCT-adjusted blood viscosity. (**c**) The square root of shear stress at the native HCT as a function of the square root of shear rate. (**d**) The square root of shear stress adjusted at 45% HCT as a function of the square root of shear rate. The dashed lines are the Casson model fits for the Control (green) and M2 (red). Power-law fits for M2 is shown as the red dotted line.

Viscoelastic properties and native-HCT WBV (η_a_) were measured at 37 °C as shown in Fig. 3a. The Control WBV was approximately 70 mPa•s at 0.1 s^−1^ and decreased to approximately 4 mPa•s at 1000 s^−1^. The shear-thinning native-HCT WBV agrees quantitatively with previous measurements for healthy subjects^11^. Previous studies also found that for normal subjects, blood viscosity exhibits a strong dependence on hematocrit. It is interesting to note the effectiveness of the hemodilution, as Fig. 3a. The native blood viscosity of M1 and M2 were very similar to the Control and M0 despite a considerably lower HCT (30% instead of 45%). This indicates that there are significant blood microstructural differences post-surgery, corresponding to larger RBC aggregates shown in Fig. 2. The minimal quantitative differences of WBV at native HCT between pre- and post-surgery illustrate the effectiveness of hemodilution treatments in lowering WBV by lowering HCT.

In contrast, significant differences in the HCT-adjusted viscosity *η*_rel(45)_ were found between M0, M1, and M2. Figure 3b shows that the *η*_rel(45)_ values for M1 and M2 were nearly an order of magnitude higher than M0 at 0.1 s^−1^, possibly due to prolonged recovery and inflammatory responses leading to increased RBC aggregation. The M3 WBV was comparable with the Control and M0, indicating that the patients’ blood rheology mostly recovered from surgery. Expectedly, the shear stress at native HCT are also very similar for different groups (Fig. 3c) despite very different HCT. However, the WBV shear-rate dependence was examined with constitutive models, as shown in Fig. 3b,d. The classical Casson model, given in Eq. 1, captures the shear-rate dependence for human whole blood^27,50,51^.

1 $${\sigma }^{1/2}={k}_{0}+{k}_{1}{\dot{\gamma }}^{1/2}$$

The viscosity dependence follows the stress dependence *η* = *σ*/$\dot{\gamma }$. Fitting to the Casson model gives the yield stress *k*_0_^2^. *k*_1_^2^, which may be interpreted as the shear-rate independent viscosity, is approximately 3 to 4 centipoises at high shear rates (greater than 100 s^−1^) for WBV. From the best fits to the data, we found that *k*_0_ = 0.0957, *k*_1_ ≈ 0.0518 (*k*_1_^2^ = 0.0027 centipoise) for the Control and *k*_0_ = 0.214, *k*_1_ ≈ 0.0449 (*k*_1_^2^ = 0.0020 centipoise) for M2. The high shear rate viscosity mainly depends on single RBC deformation, and thus *k*_1_^2^ does not vary significantly between Control and M2. However, the yield stress *k*_0_^2^ is significantly higher for M2, indicating more elastic characteristics in post-surgery subjects’ blood, likely due to larger aggregates as found in the RBC microstructure images in Fig. 2d.

The Casson model captures the stress dependence at low shear rates for the Control and the M2 groups. The model is designed to capture the extreme limits for blood. However, it can also be seen that the model does not fully capture the intermediate shear rate dependence. For $\dot{\gamma }$ > 20 s^−1^, *η*_rel(45)_ exhibits power-law dependence, *η*_rel(45)_ ~ ${\dot{\gamma }}^{n-1}$, with the exponents *n* = 0.73 (Control) and 0.57 (M2). At moderate to high shear rates, the shear thinning behavior may be strongly related to RBC deformation and shear-induced RBC depletion near the walls. To better model blood flow in this regime, these phenomena would require more detailed simulations to accurately capture the evolution of RBC microstructure and its mechanical properties.

**Figure 4:**
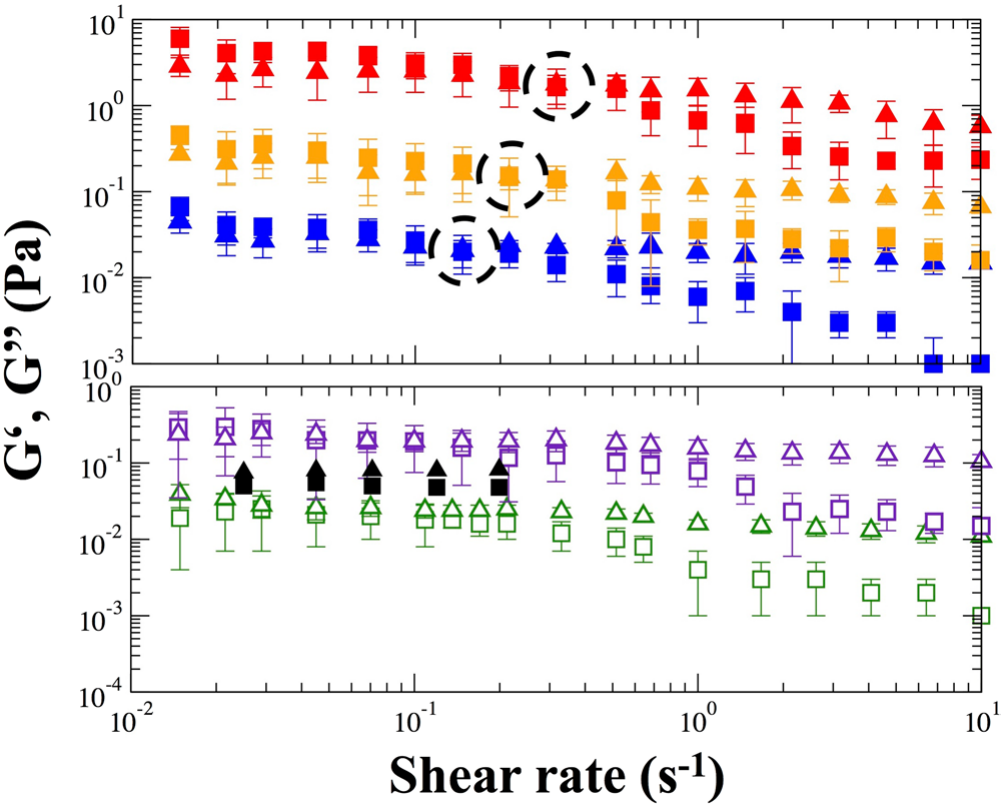
Viscoelastic behavior of blood at the native HCT. For clarification, the values of M1 and M3 are increased by 10, and the values of M2 are increased by 100. G’ (squares) and G” (triangles) from the strain amplitude oscillation sweep test for blood sample of the Control (), M0 () (${\dot{\gamma }}^{\ast }$ = 0.147 s^−1^), M1 () (${\dot{\gamma }}^{\ast }$ = 0.215 s^−1^); M2 () (${\dot{\gamma }}^{\ast }$ = 0.316 s^−1^), M3 () at a frequency of 1 rad/s, compared with five normal subjects (■▲) from previous literature^28^. Error bars indicate the interquartile (25 to 75%) range, and shear rate $(\dot{\gamma })$ = strain amplitude (*γ*)* angular frequency (*ω*).

Blood viscoelastic response was also investigated by oscillatory shear flow from $\dot{\gamma }$ = 0.01 to 10 s^−1^ by varying the strain amplitude $(\gamma =\dot{\gamma }/\omega )$ at a fixed oscillatory frequency (*ω* = 1 rad/s). The elastic (G’) and loss (G”) moduli for all groups are shown in Fig. 4. In all cases, G’ and G” decreased as strain (and shear rate) increased. Viscoelastic moduli in the low shear rate regime for the Control group are consistent with previous measurements of 5 normal subjects^28^, and we found qualitatively similar damping factor (G”/G’). Quantitative differences are attributed to differences in the oscillatory frequency, strain amplitude, and measuring geometry. For CVD subjects, the elastic character was more prominent at low strain (*G*’ > *G*”), which indicates weak solid-like or gel-like behavior. A crossover occurs at a critical strain γ*$\dot{\gamma }$* where the viscous character becomes more prominent, indicating flow and aggregate breakup. Interestingly, the crossover strain γ*(M2) > γ*(M1) > γ*(M0), suggesting blood is more gel-like post-surgery and during recovery. This is consistent with the higher low shear rate WBV for M2 and M1. In contrast, the viscous character was more prominent (G” > G’) when the strain was larger than 10% for the Control and M3 with no distinct γ*.

**Figure 5:**
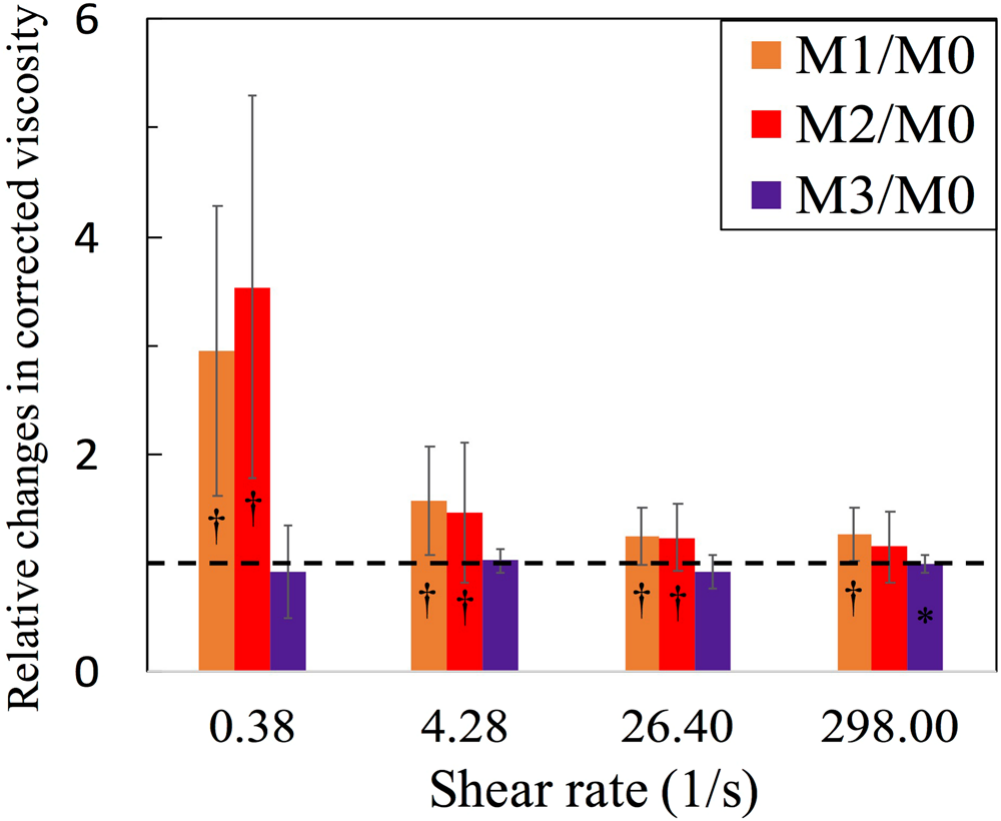
Comparison of the relative changes in *η*_rel(45)_ at four shear rates during three different periods. The value of *η*_rel(45)_ for M1, M2 and M3 is divided by M0. Error bars indicate the interquartile values, and the dashed line represents the baseline (M0/M0 = 1). “*” and “^†^”indicate *P* < 0.05 and *P* < 0.01 respectively, when compared with the baseline by the two-tailed Wilcoxon test.

To account for individual physiological differences, the relative change index (%, effect/baseline) from the pre-surgical WBV was calculated for each patient at four chosen shear rates $\dot{\gamma }$ = 0.38, 4.28, 26.4, and 298 s^−1^. Figure 5 shows significant relative changes (*P* < 0.01) for M1 and M2 for $\dot{\gamma }\,=\,0.38$, 4.28 and 26.4. For M3, WBV was statistically the same as the pre-surgery values.

Table 2 further shows that *η*_a_ was 50% higher (*P* < 0.01) for M0, M1, and M2 than in the Control at 0.38 s^−1^. The differences were very small at higher shear rates of 26.4 and 298 s^−1^. WBV shows that the low shear rate (0.38 s^−1^) *η*_rel(45)_ for M0 was 50% higher than Control (*P* < 0.01), while the HCT-adjusted WBV for M1 and M2 increased by 4- to 5-fold (both *P* < 0.01) compared to Control. Smaller but steady increases of PV were found for M0, M1, and M2 compared to Control. For M3, PV was comparable to the Control. These changes correspond to the increase of Fib and CRP as shown in Table 1.

### Correlations between blood viscosity, RBC aggregate size and fibrinogen

Even though biochemical changes are known to affect blood viscosity at low shear rates, the relationship between RBC aggregation and rheological properties remains ambiguous for CVD^52,53^. In an attempt to resolve this issue, we found two positive correlations across the different groups between (1) AAS and *η*_rel(45)_ (*r* = 0.665, P < 0.001) and (2) AAS and γ* (*r* = 0.698, P < 0.001). Although there were few quantitative differences in native-HCT WBV shear-thinning between pre- and post-surgery due to different hematocrit levels, the oscillatory shear flow measurement could differentiate the critical strains for viscoelastic behavior induced by biochemically effected changes in the inter-RBC attraction. The upshot is that RBC aggregate microstructure is sensitive to oscillatory strain at the native HCT. Moreover, the microstructure appears to be highly correlated to the increased Fib and CRP concentrations. The changes in aggregate microstructure further influence the bulk rheology of blood when comparing Fib with AAS (*r* = 0.824, P < 0.001) and *η*_rel(45)_ (*r* = 0.602, P < 0.001). Our results also indicate that aggregates are larger in size and quantity during the post-surgery inflammatory state and tend to increase blood perfusion resistance.

## Conclusion

We employed microscopy and rheological methods to investigate microstructure and rheological changes, caused by cardiac surgical procedures in CVD patients during hospitalization and long-term follow-up, over a wide range of shear rates under steady and oscillatory flow. We also examined the correlation between microstructural, rheological and biochemical assessments. Our key findings are that: *η*_rel(45)_, PV, AAS, and ASP exhibited significant changes in post-surgical patients at one day after surgery which may lead to prolonged systemic inflammatory and thrombotic responses. Although previous studies on the effect of surgery on blood properties have found that abdominal surgery patients experience slight reductions in Fib and *η*_rel(45)_ one day after surgery^54^, the dramatically changed rheological characteristics exhibited in cardiac surgical patients may be the result of a system inflammatory response to CVD surgery and possibly CPB.

With CPB, we find that hemodilution effectively controls the post-surgery blood viscosity over a wide range of shear rates. However, there remain distinctions in the viscoelastic moduli for patients as a result of larger, more rounded RBC aggregates. These differences may be characterized from the crossover strain. To reduce side effects, future treatments may consider methods for reducing and normalizing the aggregate size and shape.

We also found increased relative viscosity, low shear rate elastic modulus, larger and more randomly-shaped aggregates post-surgery. These properties were correlated with increased Fib and CRP concentrations in post-surgical subjects. These rheological properties could serve as distinct indicators for monitoring post-surgical recovery as well as identifying patients requiring surgery. These physical indicators may also be utilized to evaluate the effectiveness of treatment and recovery following cardiac surgical procedures.

Several improvements could be made to further examine blood rheological properties. Although the administrative procedure of open-heart-surgery was uniform between the various trials, many other factors, such as differences in CVD-related symptoms (diabetes, hypertension, hyperlipidemia), CVD severity, and pharmacological factors, may influence the results. In addition to this, gender and age differences between the Control and the patient groups could be improved in future studies.
